# Supplementary material for: Long-term leisure-time physical activity and other health habits as predictors of objectively monitored late-life physical activity – A 40-year twin study
Source: Sci Rep. 2018 Jun 20;8:9400. doi: 10.1038/s41598-018-27704-7 (PMC6010475; doi:10.1038/s41598-018-27704-7)
Supplement: Supplementary file 1 — Supplementary Info [file 41598_2018_27704_MOESM1_ESM.pdf]

Long-term leisure-time physical activity and other health habits as predictors of objectively monitored late-life physical activity – A 40-year twin study

Authors: Katja Waller, Henri Vähä-Ypyä, Timo Törmäkangas, Pekka Hautasaari, Noora Lindgren, Paula Iso-Markku, Kauko Heikkilä, Juha Rinne, Jaakko Kaprio, Harri Sievänen, Urho M. Kujala

Supplementary Information

Abbreviations: Abbreviations for predictor and objectively monitored physical activity variables

Supplemetary Methods and Results: Mediation analysis by quantitative trait modelling

Table S1. Daily step count by 1990 baseline covariates

Table S2. Multivariate models for LT-mMET and the other baseline predictors of moderate-to-vigorous physical activity and daily step count

Table S3. Daily step count in twin pairs discordant for different baseline characteristics

Table S4. Intraclass correlations and their 95% confidence intervals for monozygotic and dizygotic twins

Table S5. Cross-twin cross-trait correlation matrix with standard deviations on diagonal for monozygotic twins

Table S6. Cross-twin cross-trait correlation matrix with standard deviations on diagonal for dizygotic twins

Table S7. Model fit and model comparison statistics

Table S8. Standardized estimates of genetic (g^2) and environmental (e^2) components, genetic correlations (r_G) and regression coefficients (β) for midlife MET factor as risk factor of the outcome variables in best fitting models

Figure S1. Bivariate genetic model for baseline MET factor and follow-up physical activity variable

This supplementary material has been provided by the authors to give readers additional information about their work.

Abbreviations: Abbreviations for predictor and objectively monitored physical activity variables

Baseline predictor variables

LT-mMET = Leisure-time mean MET value (in MET-hours per day) to estimate the mean volume of physical activity during the three baseline survey years (from participants with complete data on physical activity in1975, 1981 and 1990)

METf = MET factor indicating leisure time MET during the baseline years from participants having leisure time physical activity data from at least one of the baseline questionnaires in 1975, 1981 and 1990

BMI = Body mass index

Follow-up objectively monitored physical activity variables

SB = Mean daily time of sedentary behavior (lying and sitting)

Standing = Mean daily time of standing

LPA = Mean daily time of light physical activity

MVPA = Mean daily time of moderate to vigorous physical activity

Steps = Mean daily step count

Peak-10min MET = Most intensive 10 minute period during the monitoring week

Supplementary Methods and Results: Mediation analysis by quantitative trait modeling

Variable transformations

For quantitative trait analysis the variables were transformed as follows. The MET variables (1975, 1981, 1990) were transformed by taking their cubic roots prior to modelling their variability as a factor. Daily step count (Steps) and mean daily time of standing (Standing) were rescaled by dividing the observed values by 1 000, mean daily time of light physical activity (LPA) by 5 000 and mean daily time of sedentary behavior (lying and sitting, SB) by 10 000. The logarithm-transformation was used for most intensive 10 minute period value during the monitoring week (Peak-10min MET) and the square root-transformation was used for mean daily time of moderate-to-vigorous physical activity (MVPA).

Quantitative trait models for the MET factor (METf) included only continuous variables and the analysis was conducted based on the maximum likelihood (ML) estimator. As smoking is a categorical variable, models including this variable were based on the weighted least squares estimator (WLS). Conventional model fit statistics were available for the ML estimator, but as the WLS is not based on maximization of the likelihood, likelihood-based indices are not available (including e.g. the information criteria). Standard errors and confidence intervals in all quantitative trait models were based on 10 000 bootstrap draws.

Intraclass correlations

Introduction to quantitative trait genetic analyses is provided elsewhere (see e.g. Neale, M.C. and Cardon, L.R. Methodology for Genetic Studies of Twins and Families. Kluwer: Dordrecht, The Netherlands [1992], and Lynch, M. and Walsh, B. Genetic Analysis of Quantitative Traits. Sinauer Associates: Sunderland, MA [1998].) and in the following we focus on the particulars concerning the present models. Table S4 shows the intraclass correlations (ICC) for monozygotic and dizygotic twins for the pooled data and for the sex groups. The ratio of the correlations can be used to assess the model variance component combinations to model in quantitative trait models (see e.g. Sham, P. Statistics in Human Genetics. Wiley: London, GB [1998]). Briefly, when the correlation ratio is equal to two exactly, then only the additive genetic variance (A) and unique environmental variance (E) can be modelled. In this case the common environmental (C) and dominance effects (D) are exactly zero, and may lead to convergence problems in estimation, if these components are modelled. When the ratio falls below two, the common environmental effect becomes non-zero; when the ratio exceeds two, the dominance genetic effect becomes non-zero. Generally, the ICC's were similar between the genders.

Bivariate models

Cross-twin cross-trait correlations are shown in Table S5 for monozygotic and Table S6 for dizygotic twins. In univariate investigations we found no significant differences between the twin pairs. Our univariate model investigations indicated that neither C- nor D- component had significant contribution on the phenotypes. We, thus, decided to model only the A and E components and their correlations in the bivariate quantitative trait models.

The quantitative trait model can be used to test, if one variable is the direct risk factor for an outcome or if the risk attributed to the outcome is mediated via genes or environmental factors. The conceptual model to test the mediating mechanism is shown in Fig. S1. As a baseline model for the test we modelled the mediation model by estimating estimates for parameters a_{12} and e_{12}, while constraining β to zero. Sub-models of the mediation model include only either the additive genetic or the unique environmental parameters. The direct risk factor model is specified by estimating the regression parameter β from the model in Fig. S1, while constraining a_{12} and e_{12} to zero and comparing model fit to the mediation model. It is also possible that there is no statistically meaningful relationship between the variables after accounting the variability by the variance components. This can be tested by setting both the dashed and dotted effects in supplement Figure 1 to zero and comparing model fit to the mediation model. In all models duplicates of twin effects including genetic effects, environmental effects, factor loadings (λ_2, λ_3) residual variations (r_1, ..., r_3), the path coefficient (β) and means were constrained equal. The model in supplement Figure 1 is usually modelled via the Cholesky decomposition model (see e.g. Neale, M.C. and Cardon, L.R. Methodology for Genetic Studies of Twins and Families. Kluwer: Dordrecht, The Netherlands [1992]). However, we used the equivalent correlated factors model to obtain direct estimates of genetic and environmental correlations (see Loehlin, J.C. The Cholesky approach: A cautionary note. *Behav. Genet*. 26, 65-69 [1996].).

Model fit

Supplementary Results

Bivariate model fit and parameter estimates

Table S7 shows model fit indices for models examining the nature of association between the MET factor and various physical activity variables. Within nested models the choice of the best fitting model was based on the sequential likelihood ratio test (LRT). If more than one candidate model remained after the LRT, the selection was based on the information criteria and residual-based criteria and parsimony so that the simplest model was chosen as the best fitting model. For step count, the genetic mediation and direct risk factor models had non-significant worsening in model fit. However, both the Akaike information criterion (AIC) and Bayesian information criterion (BIC) seemed to favour the direct risk factor model. All LRT test were non-significant for LPA and hence we preferred the model with fewest parameters, i.e. no association between the factor and LPA. For Peak-10min MET only the LRT for the genetic mediation was non-significant, indicating that the risk between these two variables seems to have largely similar genetic origin. Similar result was observed for MVPA based on the LRT and the information criteria. Based on AIC, selection of the other non-significant model, the direct risk factor model (DRF), would be 4.19×10^{-13} times as probable as the genetic factor model to minimize the information loss indicating a significantly worse model fit. So, we prefer genetic mediation model.

For standing the likelihood ratio test indicates that none of the candidate models fit has a significantly worse fit to data, and based on parsimony we conclude that there is no significant association between the variables. For sedentary behaviour only the no-association model has a significantly worse fit to the data. However, both the mediation models and the direct risk factor models have very close estimates for the information criteria. Based on AIC and BIC the direct risk factor has the lowest observed values. However, either the mediation model for r_G or r_E are 0.61 times as likely to minimize information loss, which is not significantly worse explanatory power. Hence, there is no clear evidence to favour either the mediation or direct risk factor models.

Parameter estimates from the models of supplement Table 7 are shown in Table S8. Approximately half of the variation in standing and LPA were explained by genetic factors and the remaining half by environmental factors, although there was no significant relationship to the genetic or environmental components of the MET factor. The MET factor was a direct risk factor of the sedentary behaviour and step count outcomes with standardized regression coefficients of -0.16 and 0.29, respectively. The MET factor had statistically significant genetic association with MVPA and Peak-10min MET with a genetic correlation of approximately 0.59.

Cross-trait correlation between baseline MET factor and follow-up physical activity variables was decomposed into genetic and residual parts based on the model where we estimated both the genetic and environmental correlations. For MVPA the estimated cross-trait correlation was 0.35 (95% CI: 0.25, 0.43) with approximate contribution from genetic factors: 82 (53, 100) %. For Peak-10min MET the estimated correlation was 0.34 (0.25, 0.43) with approximate contribution from genetic factors: 98 (68, 100) %.

Table S1. Daily step count by 1990 baseline covariates

		Daily step count*			R^2 (%)†	P value‡
**Body mass index**		**Normal weight (BMI <25.0)**	**Overweight (BMI =25.0 – 29.99)**	**Obese (BMI >30.00)**		
All	No. = 653<br>median<br>(95% CI)	378<br>6695<br>(6277 to 7354)	235<br>5307<br>(4746 to 5725)	40<br>3467<br>(2940 to 4857)	7.1	<0.001
Men	No. = 303<br>median<br>(95% CI)	149<br>7892<br>(6657 to 8494)	136<br>5724<br>(5117 to 6243)	18<br>4595<br>(3356 to 7882)	5.6	<0.001
Women	No. = 350<br>median<br>(95% CI)	229<br>6119<br>(5740 to 6932)	99<br>4689<br>(3987 to 5528)	22<br>3195<br>(2152 to 4857)	8.4	<0.001
**Work-related loading**		**Sedentary**	**Non-sedentary**			
All	No. = 650<br>median<br>(95% CI)	288<br>5878<br>(5570 to 6624)	362<br>6036<br>(5757 to 6406)		0.0	0.686
Men	No. = 304<br>median<br>(95% CI)	141<br>6635<br>(6127 to 7513)	163<br>6230<br>(5838 to 6768)		0.0	0.940
Women	No. = 346<br>median<br>(95% CI)	147<br>5426<br>(4875 to 5772)	199<br>5878<br>(5440 to 6250)		0.1	0.549
**Socioeconomic status**		**White collar**	**Others**			
All	No. = 605<br>median<br>(95% CI)	100<br>6689<br>(5792 to 7909)	505<br>5885<br>(5636 to 6246)		1.6	0.002
Men	No. = 285<br>median<br>(95% CI)	48<br>7713<br>(6374 to 8618)	237<br>6209<br>(5757 to 6557)		2.3	0.006
Women	No. = 320<br>median<br>(95% CI)	52<br>5649<br>(5099 to 7500)	268<br>5651<br>(5199 to 6177)		1.1	0.080
**Cigarette smoking**		**No current smoking**	**Current**			
All	No. = 654<br>median<br>(95% CI)	551<br>6230<br>(5878 to 6490)	103<br>4974<br>(3931 to 5700)		1.6	0.002
Men	No. = 304<br>median<br>(95% CI)	254<br>6605<br>(6230 to 7082)	50<br>5044<br>(3805 to 6657)		2.3	0.006
Women	No. = 350<br>median<br>(95% CI)	297<br>5754<br>(5432 to 6236)	53<br>4974<br>(3624 to 5900)		1.1	0.080
**Heavy alcohol use**		**No**	**Yes**			
All	No. = 651<br>median<br>(95% CI)	511<br>6132<br>(5830 to 6484)	140<br>5803<br>(5117 to 6243)		0.8	0.025
Men	No. = 303<br>median<br>(95% CI)	196<br>6625<br>(6209 to 7142)	107<br>6180<br>(5579 to 7096)		0.7	0.170
Women	No. = 348<br>median<br>(95% CI)	315<br>5758<br>(5463 to 6240)	33<br>5050<br>(3505 to 5651)		1.1	0.033
**Health status**		**Healthy**	**Not healthy**			
All	No. = 605<br>median<br>(95% CI)	245<br>6584<br>(6236 to 7097)	360<br>5609<br>(5239 to 5895)		2.0	0.001

**Table S1. Daily step count by 1990 baseline covariates (continues)**					
		**Daily step count***		**R^2 (%)†**	**P value‡**
Men	No. = 285<br>median<br>(95% CI)	133<br>6624<br>(6180 to 7534)	152<br>6085<br>(5292 to 6597)	2.0	0.025
Women	No. = 320<br>median<br>(95% CI)	112<br>6470<br>(5500 to 7403)	208<br>5450<br>(4974 to 5758)	2.1	0.018

*Descriptive analyses with bootstrapping (1000 samples)
†R^2 for each baseline variable calculated as a difference (ΔR^2) from age and sex model compared to model with variable (e.g. bmi90) + age and sex, indicating the true R^2 of the studies variable
‡P value from linear regression adjusted for sex and age and cluster for family

Table S2. Multivariate models for LT-mMET and the other baseline predictors of moderate-to-vigorous physical activity and daily step count

	**Moderate to vigorous physical activity**											
	Model 1*			Model 2†			Model 3‡			Model 4§		
	No.	R^2 (%)	P value	No.	R^2 (%)	P value	No.	R^2 (%)	P value	No.	R^2 (%)	P value
All	596	8.4	<0.001	596	17.2	<0.001	596	18.7	<0.001	596	20.3	<0.001
Men	279	8.7	<0.001	279	13.5	<0.001	279	16.5	<0.001	279	19.2	<0.001
Women	317	6.1	<0.001	317	19.0	0.001	317	19.7	0.002	317	20.7	0.004
	**Daily step count**											
	Model 1*			Model 2†			Model 3‡			All§		
	No.	R^2 (%)	P value	No.	R^2 (%)	P value	No.	R^2 (%)	P value	No.	R^2 (%)	P value
All	596	6.3	<0.001	596	12.1	<0.001	596	13.9	<0.001	596	15.0	<0.001
Men	279	7.3	<0.001	279	10.7	<0.001	279	14.0	0.001	279	15.1	0.001
Women	317	4.8	<0.001	317	12.6	0.001	317	13.5	0.002	317	14.8	0.006

*Model 1 including sex, age and LT-mMET
†Model 2 including sex, age, body mass index and LT-mMET
‡Model 3 including sex, age, body mass index, cigarette smoking and LT-mMET
§Model 4 including sex, age, body mass index, cigarette smoking, socioeconomic status, health status and LT-mMET
P values are for the statistical significance of the LT-mMET in each model.

Table S3. Daily step count in twin pairs discordant for different baseline characteristics

	No.		Daily step count*		Z and P value[‡]
**LT-mMET**			**Lower LT-mMET**	**Higher LT-mMET**	
All twin pairs	23	median (IQR)<br>(95% CI)	5099 (3919)<br>(4197 to 7513)	7142 (5060)<br>(4697 to 8886)	Z = 0.791<br>P = 0.429
DZ twin pairs	13	median (IQR)<br>(95% CI)	4846 (3980)<br>(4197 to 7513)	7142 (4874)<br>(5260 to 9440)	Z = 1.503<br>P = 0.133
MZ twin pairs	10	median (IQR)<br>(95% CI)	6970 (4075)<br>(3999 to 8052)	6442 (5694)<br>(3680 to 9110)	Z = 0.663<br>P = 0.508
**Body mass index[§]**			**Lower BMI**	**Higher BMI**	
All twin pairs	55	median (IQR)<br>(95% CI)	6584 (5080)<br>(5220 to 7406)	4950 (4806)<br>(3633 to 5873)	Z = 1.542<br>P = 0.123
DZ twin pairs	37	median (IQR)<br>(95% CI)	6711 (5106)<br>(5782 to 8293)	4056 (4804)<br>(3361 to 6274)	Z = 2.165<br>P = 0.030
MZ twin pairs	15	median (IQR)<br>(95% CI)	5129 (5981)<br>(1932 to 7406)	4950 (5269)<br>(2650 to 6653)	Z = 0.625<br>P = 0.532
**Work-related loading**			**Sedentary**	**Non-sedentary**	
All twin pairs	77	median (IQR)<br>(95% CI)	6742 (3912)<br>(5890 to 7830)	5958 (4205)<br>(5135 to 6936)	Z = 1.160<br>P = 0.246
DZ twin pairs	45	median (IQR)<br>(95% CI)	6711 (3570)<br>(5782 to 7810)	5900 (4610)<br>(4846 to 6772)	Z = 1.157<br>P = 0.247
MZ twin pairs	29	median (IQR)<br>(95% CI)	7754 (4143)<br>(5767 to 8519)	6114 (3698)<br>(5129 to 7951)	Z = 0.400<br>P = 0.689
**Socioeconomic status**			**White collar**	**Others**	
All twin pairs	24	median (IQR)<br>(95% CI)	6447 (4343)<br>(5792 to 8434)	6240 (3080)<br>(5601 to 7207)	Z = 0.914<br>P = 0.361
DZ twin pairs	17	median (IQR)<br>(95% CI)	6248 (4139)<br>(5199 to 8127)	6243 (3505)<br>(5120 to 7384)	Z = 0.497<br>P = 0.619
MZ twin pairs	7	median (IQR)<br>(95% CI)	8434 (4822)<br>(5376 to 10859)[b]	6236 (3283)<br>(4808 to 8745)[†]	Z = 1.014<br>P = 0.310
**Cigarette smoking**			**No current smoking**	**Current**	
All twin pairs	40	median (IQR)<br>(95% CI)	5864 (4548)<br>(5337 to 7679)	4598 (5601)<br>(3552 to 6408)	Z = 2.083<br>P = 0.037
DZ twin pairs	21	median (IQR)<br>(95% CI)	5642 (4110)<br>(4159 to 7545)	5281 (4746)<br>(3366 to 6660)	Z = 0.991<br>P = 0.322
MZ twin pairs	15	median (IQR)<br>(95% CI)	8409 (4996)<br>(4925 to 9539)	4693 (5717)<br>(3168 to 8507)	Z = 1.931<br>P = 0.053
**Heavy alcohol use**			**No**	**Yes**	
All twin pairs	36	median (IQR)<br>(95% CI)	6422 (4346)<br>(5349 to 8101)	6073 (3986)<br>(5099 to 8127)	Z = 0.094<br>P = 0.925
DZ twin pairs	22	median (IQR)<br>(95% CI)	5920 (4517)<br>(4865 to 7788)	6073 (3989)<br>(5099 to 8127)	Z = -0.503<br>P = 0.615
MZ twin pairs	13	median (IQR)<br>(95% CI)	7097 (3895)<br>(5266 to 9157)	6570 (4426)<br>(4092 to 8508)	Z = -1.083<br>P = 0.279
**Health status**			**Healthy**	**Not healthy**	
All twin pairs	69	median (IQR)<br>(95% CI)	6767 (4542)<br>(5838 to 8236)	5900 (4490)<br>(5186 to 6788)	Z = 2.042<br>P = 0.041
DZ twin pairs	37	median (IQR)<br>(95% CI)	6711 (5159)<br>(5724 to 8569)	5474 (4484)<br>(4607 to 7647)	Z = 1.622<br>P = 0.105
MZ twin pairs	26	median (IQR)<br>(95% CI)	6720 (4196)<br>(5405 to 8511)	6625 (3876)<br>(5440 to 8527)	Z = 0.140<br>P = 0.889

*All analyses with bootstrapping (1000 samples unless otherwise noted)
[†]Bootstrap based on 994 samples
[‡]Z as absolute value and P by Wilcoxon matched-pairs signed-rank test
[§]BMI difference ≥3 between twin pairs when at least one twin is overweight (BMI ≥25)

Table S4. Intraclass correlations and their 95% confidence intervals (CIs) for monozygotic and dizygotic twins

	All		Men		Women	
	ICC (95% CI)		ICC (95% CI)		ICC (95% CI)	
	MZ	DZ	MZ	DZ	MZ	DZ
SB	0.41 (0.25, 0.55)	0.16 (0.00, 0.31)	0.49 (0.27, 0.66)	0.16 (-0.07, 0.38)	0.34 (0.10, 0.54)	0.16 (-0.07, 0.37)
Standing	0.49 (0.34, 0.61)	0.16 (0.00, 0.31)	0.55 (0.35, 0.71)	0.09 (-0.14, 0.31)	0.39 (0.16, 0.58)	0.21 (-0.02, 0.41)
LPA	0.48 (0.33, 0.60)	0.40 (0.26, 0.53)	0.38 (0.14, 0.58)	0.37 (0.16, 0.55)	0.55 (0.35, 0.70)	0.44 (0.24, 0.61)
MVPA	0.57 (0.44, 0.68)	0.28 (0.13, 0.43)	0.53 (0.31, 0.69)	0.17 (-0.06, 0.38)	0.60 (0.42, 0.74)	0.37 (0.16, 0.55)
Steps	0.51 (0.37, 0.63)	0.27 (0.11, 0.41)	0.49 (0.27, 0.67)	0.22 (-0.01, 0.42)	0.51 (0.30, 0.67)	0.31 (0.09, 0.50)
Peak-10min MET	0.59 (0.46, 0.69)	0.20 (0.04, 0.35)	0.53 (0.32, 0.69)	0.15 (-0.08, 0.36)	0.64 (0.47, 0.77)	0.24 (0.02, 0.44)
METf*	0.44 (0.07, 0.94)	0.22 (0.04, 0.60)	0.11 (-0.11, 0.90)	0.07 (-0.19, 0.49)	0.91 (0.48, 1.18)	0.71 (0.13, 0.99)

*Confidence intervals based on 10 000 bootstrap draws

Table S5. Cross-twin cross-trait correlation matrix with standard deviations on diagonal for monozygotic twins

	SB1	Standing1	LPA1	MVPA1	Steps1	Peak-10min MET1	METf1	SB2	Standing2	LPA2	MVPA2	Steps2	Peak-10min MET2	METf2
SB1	0.54													
Standing1	**-0.48**	2.61												
LPA1	**-0.42**	0.07	0.69											
MVPA1	**-0.31**	0.02	0.16	17.2										
Steps1	**-0.35**	0.09	**0.35**	**0.84**	3.07									
Peak-10min MET1	**-0.34**	0.08	0.12	**0.88**	**0.75**	0.24								
METf1	**-0.17**	-0.02	**0.24**	**0.46**	**0.42**	**0.37**	1.42							
SB2	**0.41**	**-0.21**	**-0.28**	**-0.17**	**-0.21**	**-0.21**	-0.09	0.61						
Standing2	**-0.21**	**0.48**	0.13	**0.18**	**0.22**	**0.21**	0.04	**-0.44**	2.67					
LPA2	-0.08	0.05	**0.48**	0.01	0.10	0.01	0.18	**-0.50**	**0.28**	0.71				
MVPA2	**-0.21**	0.11	0.16	**0.57**	**0.50**	**0.56**	**0.25**	**-0.35**	**0.19**	0.09	16.3			
Steps2	**-0.20**	0.13	**0.24**	**0.49**	**0.50**	**0.49**	**0.21**	**-0.40**	**0.29**	**0.22**	**0.87**	3.21		
Peak-10min MET2	**-0.20**	0.17	0.09	**0.56**	**0.46**	**0.59**	**0.27**	**-0.31**	**0.25**	0.00	**0.88**	**0.73**	0.24	
METf2	-0.12	0.03	0.09	**0.37**	**0.40**	**0.36**	0.56	-0.14	**0.18**	-0.06	**0.21**	**0.22**	**0.22**	1.76

Note. Bold typeface indicates statistically significant at 0.05 level of significance

Table S6. Cross-twin cross-trait correlation matrix with standard deviations on diagonal for dizygotic twins

	SB1	Standing1	LPA1	MVPA1	Steps1	Peak-10min MET1	METf1	SB2	Standing2	LPA2	MVPA2	Steps2	Peak-10min MET2	METf2
SB1	0.62													
Standing1	**-0.38**	2.43												
LPA1	**-0.57**	**0.21**	0.78											
MVPA1	**-0.39**	**0.21**	**0.18**	18.4										
Steps1	**-0.46**	**0.29**	**0.36**	**0.83**	3.26									
Peak-10min MET1	**-0.35**	**0.25**	**0.14**	**0.90**	**0.77**	0.27								
METf1	-0.14	0.09	0.11	**0.31**	**0.29**	**0.34**	1.42							
SB2	**0.16**	-0.07	**-0.27**	-0.07	-0.15	-0.07	-0.08	0.66						
Standing2	-0.00	**0.15**	0.06	0.04	0.04	0.06	0.09	**-0.52**	2.94					
LPA2	**-0.16**	-0.01	0.38	0.02	0.07	-0.02	-0.01	**-0.63**	**0.25**	0.78				
MVPA2	-0.03	0.08	-0.00	**0.28**	**0.30**	**0.20**	0.13	**-0.33**	**0.32**	**0.19**	18.0			
Steps2	-0.03	0.06	0.08	**0.22**	**0.27**	0.14	0.03	**-0.47**	**0.37**	**0.39**	**0.85**	3.01		
Peak-10min MET2	0.03	0.06	-0.06	**0.24**	**0.25**	**0.19**	**0.22**	**-0.31**	**0.37**	**0.15**	**0.88**	**0.77**	0.25	
METf2	-0.05	0.01	0.17	0.13	0.16	0.15	**0.39**	-0.12	0.14	0.04	**0.31**	**0.32**	**0.35**	1.76

Note. Bold typeface indicates statistically significant at 0.05 level of significance

Table S7. Model fit and model comparison statistics

		Model fit							Model comparison			
			LRT		Residual-based		Incremental fit		LRT		Information criteria	
	**Model**	**LL**	**χ^2 (df)**	**p**	**RMSEA (90% CI)**	**SRMR**	**CFI**	**TLI**	**χ^2 (df)**	**p**	**AIC**	**BIC**
SB	r_G, r_E	-1707	69.9 (73)	0.582	0.00 (0.00, 0.03)	0.12	1.00	1.00			3444	3506
	r_G, --	-1708	71.6 (74)	0.556	0.00 (0.00, 0.04)	0.12	1.00	1.00	1.75 (1)	0.186	3443	3502
	--, r_E	-1708	71.4 (74)	0.565	0.00 (0.00, 0.04)	0.12	1.00	1.00	1.49 (1)	0.222	3443	3501
	--, --	-1713	82.8 (75)	0.252	0.02 (0.00, 0.04)	0.13	0.98	0.98	12.9 (2)	0.002	3453	3506
	DRF	-1707	69.9 (74)	0.614	0.00 (0.00, 0.03)	0.12	1.00	1.00	0.01 (1)	0.944	3442	3500
Standing	r_G, r_E	-2798	69.3 (73)	0.600	0.00 (0.00, 0.03)	0.09	1.00	1.00			5627	5689
	r_G, --	-2799	70.0 (74)	0.612	0.00 (0.00, 0.03)	0.09	1.00	1.00	0.61 (1)	0.434	5625	5684
	--, r_E	-2798	69.6 (74)	0.624	0.00 (0.00, 0.03)	0.09	1.00	1.00	0.62 (1)	0.623	5625	5683
	--, --	-2800	72.2 (75)	0.571	0.00 (0.00, 0.03)	0.09	1.00	1.00	0.24 (2)	0.241	5626	5680
	DRF	-2798	69.4 (74)	0.631	0.00 (0.00, 0.03)	0.09	1.00	1.00	0.86 (1)	0.858	5625	5683
LPA	r_G, r_E	-1847	77.5 (73)	0.337	0.02 (0.00, 0.04)	0.09	0.99	0.99			3724	3786
	r_G, --	-1848	78.9 (74)	0.328	0.02 (0.00, 0.04)	0.09	0.99	0.99	1.36 (1)	0.244	3723	3782
	--, r_E	-1847	77.5 (74)	0.367	0.01 (0.00, 0.04)	0.09	0.99	0.99	0.02 (1)	0.885	3722	3780
	--, --	-1848	79.6 (75)	0.335	0.02 (0.00, 0.04)	0.09	0.99	0.99	2.14 (2)	0.342	3722	3776
	DRF	-1847	78.0 (74)	0.353	0.02 (0.00, 0.04)	0.09	0.99	0.99	0.50 (1)	0.482	3722	3781
MVPA	r_G, r_E	-4158	65.0 (73)	0.738	0.00 (0.00, 0.03)	0.09	1.00	1.00			8346	8408
	r_G, --	-4159	66.5 (74)	0.719	0.00 (0.00, 0.03)	0.09	1.00	1.00	1.57 (1)	0.210	8345	8404
	--, r_E	-4168	84.6 (74)	0.187	0.03 (0.00, 0.05)	0.11	0.98	0.98	19.7 (1)	<0.001	8364	8422
	--, --	-4188	125.2 (75)	<0.001	0.05 (0,04,0,07)	0.13	0.88	0.91	60.3 (2)	<0.001	8402	8456
	DRF	-4160	68.4 (74)	0.662	0.00 (0.00, 0.03)	0.09	1.00	1.00	3.42 (1)	0.064	8347	8405

r_G: genetic correlation, r_E: environmental correlation, DRF: direct risk factor, LRT: likelihood ratio test, LL: log-likelihood, RMSEA: root mean square error of approximation, SRMR: standardized root mean square residual, CFI: comparative fit index, TLI: Tucker-Lewis index, AIC: Akaike information criterion, BIC: Bayesian information criterion.

Table S7. Model fit and model comparison statistics (continued)

		Model fit							Model comparison			
			LRT		Residual-based		Incremental fit		LRT		Information criteria	
	Model	LL	χ^2 (df)	p	RMSEA (90% CI)	SRMR	CFI	TLI	χ^2 (df)	p	AIC	BIC
Steps	r_G, r_E	-2889	59.3 (73)	0.876	0.00 (0.00, 0.02)	0.08	1.00	1.00			5807	5870
	r_G, --	-2890	61.6 (74)	0.848	0.00 (0.00, 0.02)	0.08	1.00	1.00	2.25 (1)	0.133	5808	5866
	--, r_E	-2894	70.9 (74)	0.581	0.00 (0.00, 0.03)	0.10	1.00	1.00	11.6 (1)	0.001	5817	5875
	--, --	-2910	102.8 (75)	0.018	0.04 (0.02, 0.06)	0.12	0.93	0.95	43.4 (2)	<0.001	5847	5901
	DRF	-2889	60.4 (74)	0.873	0.00 (0.00, 0.02)	0.08	1.00	1.00	1.07 (1)	0.301	5806	5865
Peak-10min MET	r_G, r_E	-1001	64.7 (73)	0.744	0.00 (0.00, 0.03)	0.11	1.00	1.00			2033	2095
	r_G, --	-1001	64.8 (74)	0.770	0.00 (0.00, 0.03)	0.11	1.00	1.00	0.02 (1)	0.890	2031	2089
	--, r_E	-1015	91.0 (74)	0.087	0.03 (0.00, 0.05)	0.13	0.96	0.97	26.3 (1)	<0.001	2057	2115
	--, --	-1031	123.8 (75)	<0.001	0.05 (0.04, 0.07)	0.15	0.88	0.91	59.0 (2)	<0.001	2088	2142
	DRF	-958	70.6 (74)	0.591	0.00 (0.00, 0.03)	0.13	1.00	1.00	5.86 (1)	0.016	1944	2002

r_G: genetic correlation, r_E: environmental correlation, DRF: direct risk factor, LRT: likelihood ratio test, LL: log-likelihood, RMSEA: root mean square error of approximation, SRMR: standardized root mean square residual, CFI: comparative fit index, TLI: Tucker-Lewis index, AIC: Akaike information criterion, BIC: Bayesian information criterion.

Table S8. Standardized estimates of genetic (g^2) and environmental (e^2) components, genetic correlations (r_G) and regression coefficients (β) for baseline MET factor as risk factor of the outcome variables in best fitting models

		MET factor components		Outcome components			
**Outcome**	**Model**	g^2	e^2	g^2	e^2	r_G	β
SB	DRF	0.54 (0.25, 0.78)	0.46 (0.22, 0.75)	0.41 (0.24, 0.56)	0.59 (0.44, 0.77)	--	-0.16 (-0.26, -0.06)
Standing	--, --	0.54 (0.24, 0.79)	0.46 (0.22, 0.76)	0.45 (0.32, 0.56)	0.55 (0.44, 0.68)	--	--
LPA	--, --	0.54 (0.24, 0.79)	0.46 (0.22, 0.76)	0.56 (0.42, 0.67)	0.44 (0.33, 0.58)	--	--
MVPA	r_G, --	0.55 (0.29, 0.78)	0.45 (0.22, 0.71)	0.60 (0.49, 0.70)	0.40 (0.30, 0.51)	0.59 (0.44, 0.79)	--
Steps	DRF	0.58 (0.32, 0.78)	0.42 (0.22, 0.68)	0.48 (0.35, 0.60)	0.52 (0.40, 0.65)	--	0.29 (0.19, 0.38)
Peak-10min MET	r_G, --	0.57 (0.33, 0.79)	0.43 (0.21, 0.67)	0.60 (0.45, 0.73)	0.40 (0.27, 0.55)	0.59 (0.44, 0.78)	--

Note. Unstandardized risk coefficient and its 95% confidence interval in the direct risk factor model for SB: -0.37 (-0.60, -0.14) and Steps: 3.35 (2.23, 4.54). Confidence intervals based on 10 000 bootstrap draws. g^2 indicates the broad sense heritability estimate.

Figure S1. Bivariate genetic model for baseline MET factor (METf)) and follow-up physical activity variable (PA)

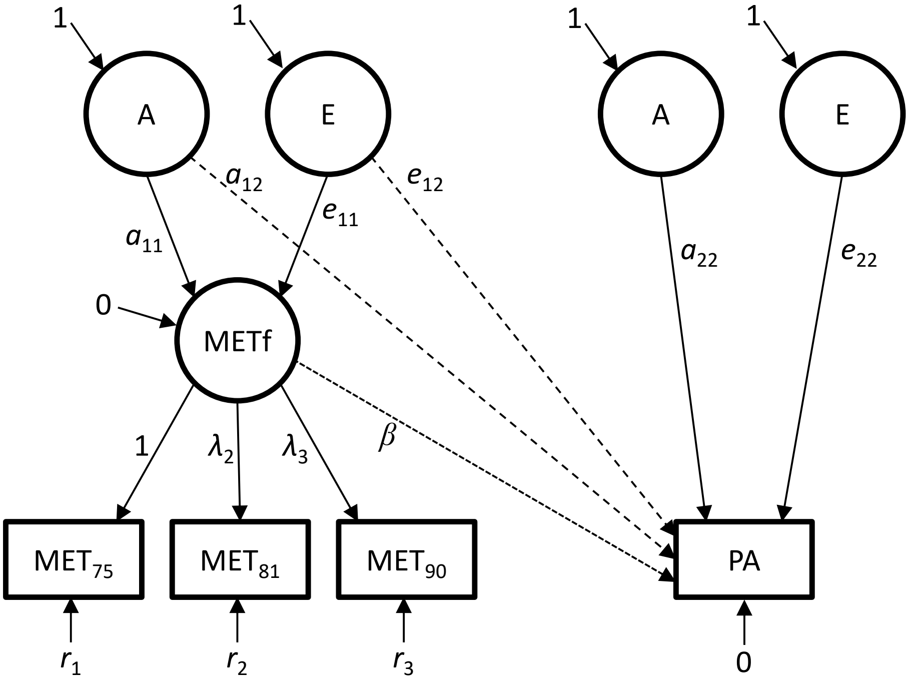

By modelling the effects shown with dashed lines, the parameters of the gene-environment mediation model can be estimated. The direct risk factor effect is shown by the dotted line.
